# Explainable graph neural networks for organic cages†

**DOI:** 10.1039/d1dd00039j

**Published:** 2022-02-11

**Authors:** Qi Yuan, Filip T. Szczypiński, Kim E. Jelfs

**Affiliations:** Department of Chemistry, Molecular Sciences Research Hub, White City Campus, Imperial College London Wood Lane London UK k.jelfs@imperial.ac.uk

## Abstract

The development of accurate and explicable machine learning models to predict the properties of topologically complex systems is a challenge in materials science. Porous organic cages, a class of polycyclic molecular materials, have potential application in molecular separations, catalysis and encapsulation. For most applications of porous organic cages, having a permanent internal cavity in the absence of solvent, a property termed “shape persistence” is critical. Here, we report the development of Graph Neural Networks (GNNs) to predict the shape persistence of organic cages. Graph neural networks are a class of neural networks where the data, in our case that of organic cages, are represented by graphs. The performance of the GNN models was measured against a previously reported computational database of organic cages formed through a range of [4 + 6] reactions with a variety of reaction chemistries. The reported GNNs have an improved prediction accuracy and transferability compared to random forest predictions. Apart from the improvement in predictive power, we explored the explicability of the GNNs by computing the integrated gradient of the GNN input. The contribution of monomers and molecular fragments to the shape persistence of the organic cages could be quantitatively evaluated with integrated gradients. With the added explicability of the GNNs, it was possible not only to accurately predict the property of organic materials, but also to interpret the predictions of the deep learning models and provide structural insights for the discovery of future materials.

## Abstract 2

We report the development of explainable Graph Neural Networks to predict shape persistence of organic cages. Integrated gradient analysis identifies collapse-inducing molecular fragments and helps chemists design more shape persistent structures.

## Graphical abstract

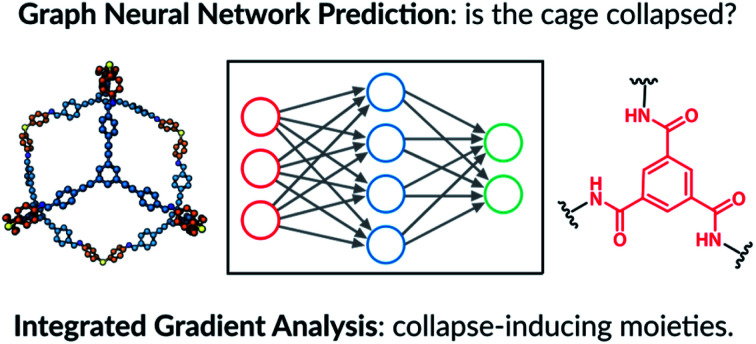

## 1. Introduction

Porous organic cages are a class of molecules with an internal cavity that is made accessible to guest molecules *via* at least two molecular windows (“intrinsic porosity”).^1,2^ Poor packing of large organic cages in the solid-state results in accessible channels between the individual molecules (“extrinsic porosity”). The cavity of porous organic cages offers potential applications including encapsulation,^3^ molecular separation,^4–7^ and catalysis.^8^ Thanks to their molecular structure, organic cages are usually soluble in organic solvents, allowing for solution processing into thin films or membranes both in the crystalline and amorphous solid state.^9^ Unlike other porous materials such as zeolites and metal–organic frameworks (MOFs), cages lack an extended network of bonds in the solid state. The absence of three-dimensional chemical bonding allows the solid-state structures to undergo large rearrangements between polymorphs, which has been used in the creation of molecular crystals exhibiting “on/off” extrinsic porosity switching.^10^ However, such flexibility also means that individual cage molecules are more likely to collapse and lose their intrinsic porosity as a result of desolvation,^11^ which is known as a lack of “shape persistence”. The shape persistence of organic cages is difficult to predict without employing computational modelling.^11,12^ High-throughput computational screening has been used in combination with robotic synthesis for the discovery of novel organic cages, but the number of structures reported experimentally is still relatively low, especially compared to isoreticular MOFs.^12^ The cost of computational screening of organic cages is significantly cheaper than experimental measurements, however modelling larger systems requires specialist software and is still time consuming, especially for organic cages that often have several hundred atoms.

Machine learning (ML) has many potential uses within material discovery, including to reduce the cost of property calculation compared to carrying out computational simulations (especially *via* quantum mechanical methods) and to remove the need for specialist modelling packages. This allows researchers to focus experimental synthesis and measurement effort on the most promising materials, reducing wasted laboratory resources,^13,14^ as well as to help facilitate the exploration of larger chemical space.^15–17^ Apart from the widely reported ML models for molecular discovery, especially drug discovery, the applications of ML to porous materials such as MOFs have gained significant interest.^18^ Various structural and geometrical descriptors for MOFs have been developed for the prediction of their gas sorption,^19^ and open-source databases recording the structures with experimental and/or computational properties have been published and the diversity of the chemical space has been examined.^20^ The development and application of ML to modelling the properties of organic cages, on the other hand, is less reported.

We have previously developed a computational database of >60 000 organic cages formed through a range of reaction chemistries *via* a [4 + 6] reaction of four tritopic and six ditopic building blocks and studied their behaviour using molecular dynamics (MD) simulations.^21^ Each cage was allowed to evolve at an elevated temperature to sample different regions of the conformational potential energy surface. A subset of structures along the MD trajectory was then optimised and the resulting lowest energy conformations were analysed for shape persistence, following heuristic rules based on the expected number of windows and the observed window diameters. We then modelled the computed shape persistence of these cages using random forest models with Morgan fingerprints – vectors indicating the presence of specific substructures within a molecule – as the input features to the model. We found the models to be very effective when applied to systems with the same reaction chemistry, for example a random forest model trained on cages formed from imine chemistry was effective at predicting shape persistence in other imine cages.^21^ However, the random forest model did not translate well between cages formed from different reaction mechanisms: an imine-trained random forest model was not as effective at predicting the shape persistence of a cage formed by alkyne metathesis chemistry. This was not surprising given that experimentally, extremely small changes to the synthesis, for example adding a single CH_2_ group to one cage precursor, could completely invert the shape persistence behaviour.^11^ The prediction result of the random forest models could not be attributed to specific monomers of the cage or fragments of the monomers, because the feature importance analysis did not show a strong preference to any specific molecular features.^21^ Recently developed graph neural networks (GNNs), which encode molecular information into neural graph fingerprints with machine-learned continuous numeric vector representation, have exhibited improved predictive performance on various tasks including chemical reactivity,^22^ compound protein interaction^23^ and partial charge assignment,^24^ because of the flexibility of such fingerprints, especially when a larger dataset is available.^25^

An additional benefit of prediction *via* GNNs is that it is possible to identify key building blocks or molecular fragments contributing to the models' predictions through calculating attribution scores of the input features. Sundararajan *et al.* developed the integrated gradients approach to compute the contribution of input features for ML tasks and highlighted a case study of explaining molecular binding mechanisms using integrated gradients.^26^ McCloskey *et al.* calculated the attribution score of fragments of molecules with a hypothesised binding mechanism and proposed a sanity check to determine whether a hypothesised mechanism can be learned.^27^ The explicability of ML models for predictive tasks in material and molecular discovery has gained increasing research interest, since explainable models can not only provide insight for the monomers and fragments that contribute exclusively to the prediction to help future discovery, but also suggest possible pitfalls of the models where predictions are accurate, but the underlying chemical mechanism has not been learnt.

In this study, we developed GNN models to predict the shape persistence of organic cages formed *via* different [4 + 6] reaction chemistries: imine condensation, amide condensation, and alkene/alkyne metathesis. Graph representations of the organic cages were developed and neural fingerprints for cages were trained using the GNN architecture. The modular structure of organic cages allows us to represent the cage structure purely by combining separate representations of the building blocks and linkers. In this way, the entire connectivity information is represented more simply and avoids redundancy present if the entire polycyclic macromolecular cage graph were used. Furthermore, this approach allows us to easily create representations for cages – be it by synthetic end-users or for future development – that use different condensation chemistries from already existing precursors. The shape persistence of the organic cages was accurately predicted using the GNN model, with significant improvement of generalisability towards unseen monomers compared to prior work with random forest models. In addition, to obtain explicability of the prediction of the GNNs, the integrated gradient was implemented and computed for precursors of the organic cages and fragments of the precursors. It was therefore possible to quantify the contribution of precursors as well as fragments to the shape persistence of organic cages and provide insight for the design of future precursors for organic cages.

## 2. Methods

### 2.1 Dataset

**Fig. 1:**
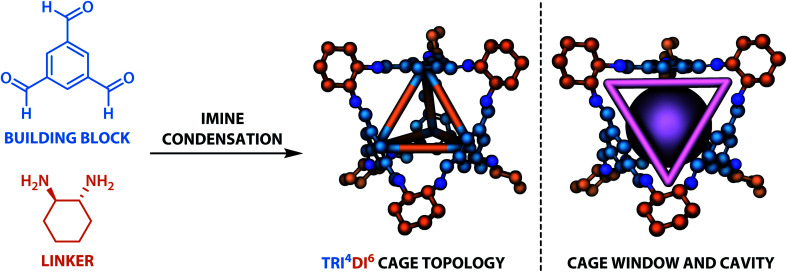
The Tri^4^Di^6^ tetrahedral topology of the organic cages considered in this study. The tritopic precursor (“building block”) is shown in blue and the ditopic precursor (“linker”) in orange. The resulting cavity and one of the four windows are highlighted in purple (right). In 3D models, hydrogen atoms are omitted for clarity, carbon atoms are based on whether they originate from the building block (blue) or the precursor (orange), nitrogen atoms are shown in dark blue.

The dataset for organic cages used here was reported in our previous work.^21^ In brief, the synthetically viable library of di- and tritopic precursors was generated based on synthetic experience, and 118 di-topic and 51 tritopic precursor cores were included, each with locations of functional groups marked. In this work, the precursors with the greater number of reactive functional groups are referred to as the “building block”, and the precursors with fewer functional groups are referred as the “linker”. Each precursor backbone was expanded with different functional groups to include organic cages synthesised with different reaction chemistry. The functional groups included were aldehydes, alkynes, amines, carboxylic acids, alkenes, which are combined using imine or amide condensation, alkyne or alkene metathesis, and disulfide formation reactions. The topologies of organic cages were defined previously by Santolini *et al.*^28^ Here, we used only the Tri^4^Di^6^ cages assembled from four tritopic precursors and six ditopic precursors in a [4 + 6] reaction (example cage in Tri^4^Di^6^ topology is shown in Fig. 1), and the previously reported random forest models were used as a benchmark for our work. For each pair of functional groups capable of undergoing a reaction, every possible pair of precursors was used to generate a cage. For each reaction, 6018 distinct precursor pairs were generated, resulting in a total of 36 108 cages. A summary of the precursor pairing for the Tri^4^Di^6^ cages is shown in Table 1.

**Table 1:** Groups of investigated Tri^4^Di^6^ cages in this study, together with the corresponding precursor scaffolds and reaction types. Each group contains 6018 structures. The number following a functional group name indicates the topicity of the precursor

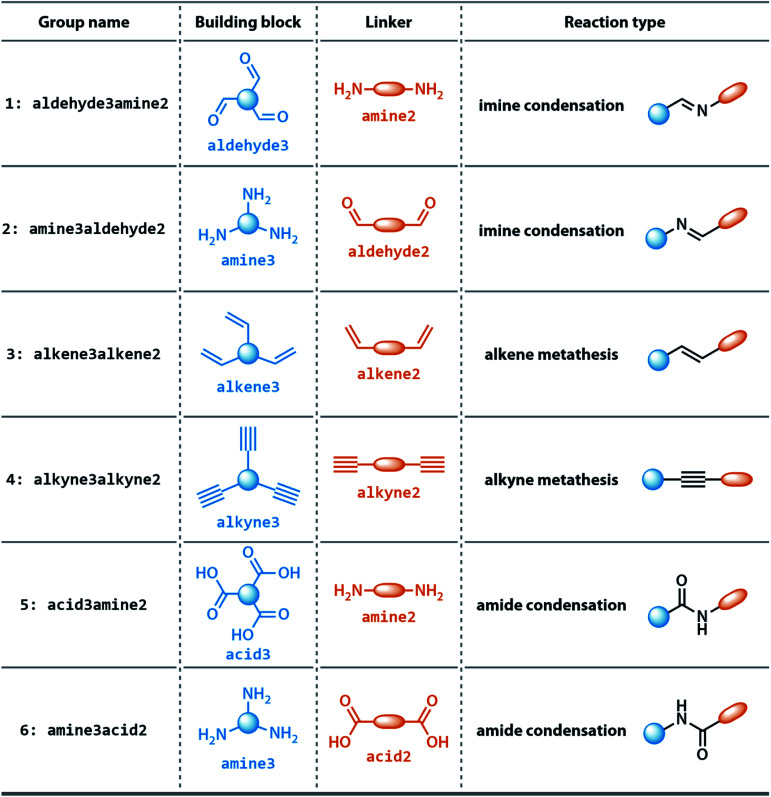

### 2.2 Dataset labelling

The same computational labels of shape persistence for the [4 + 6] organic cages published previously^21^ were used in this work, where the shape persistence of the cages was calculated from the geometrically optimised structures. Specifically, the conformational potential energy surface of the organic cages was sampled using high temperature MD simulations (2 ns at 700 K using the OPLS3 force field), followed by Polak–Ribière conjugate gradient minimisation of 50 structures selected at regular intervals along the MD trajectory. The cavity size, window diameter and number of windows of the resulting lowest energy structure for each cage were calculated using pywindow,^29^ and the cages were labelled as either “collapsed”, “not collapsed” (*i.e.*, shape persistent), or “undetermined” using the above parameters. If the cages did not contain the expected four windows for a tetrahedral topology, the cage was labelled as “collapsed”. For cages with the expected number of windows detected by pywindow, the following empirical criterion was applied:1
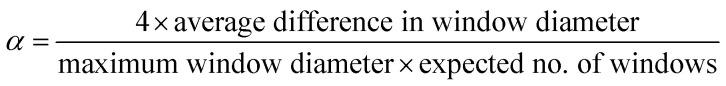


If *α* < 0.035 and the cavity size was greater than 1 Å, the cage was labelled as “not collapsed”, else it was labelled “undetermined”. Only the “not collapsed” (four windows and *α* < 0.035) and “collapsed” (wrong number of windows) cages were used to train the ML models in this study. There are 16 921 “collapsed” cages (46.8%), 12 167 “not collapsed” cages (33.7%) and 7020 “undetermined” cages in the database. Summary statistics of cage collapse labels for different chemical reactions in this study are provided in Table S1.† An example cavity with the corresponding window can be seen on the right of Fig. 1.

### 2.3 Representation of cages

**Fig. 2:**
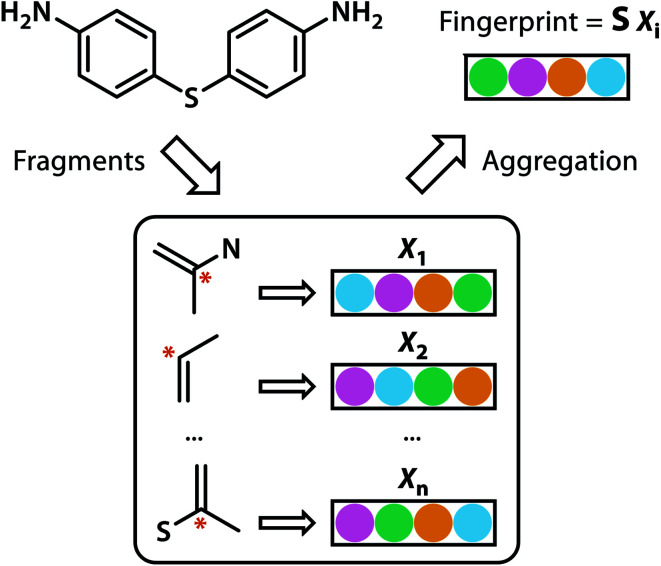
GNN encoding of the molecular features of the building blocks and linkers of organic cages in this study.

Building blocks and linkers of the organic cages were encoded using the graph neural network (GNN), where representation of each atom in the molecule was obtained by aggregating the information of the atom and its neighbours. The design of the GNN layer for encoding the building blocks and linkers is shown in Fig. 2. Each non-hydrogen atom *X* in the molecule was initialised using a numeric vector in the form of *X*_i_ = (*V*_atom_,*V*_neighbour_,*V*_2nd neighbour_). *V*_atom_ was calculated using RDkit^30^ and contains information including atomic symbol, number of neighbour non-hydrogen atoms, implicit and explicit valence, and whether the atom is aromatic. *V*_neighbour_ is the sum of the atomic vector of the atom and its neighbours weighted by the bond order. *V*_2nd neighbour_ contains the sum of the atomic vector of the atom and up to its second-degree neighbour weighted by the bond order. Given that *V*_atom_ is not scaled, while *V*_neighbour_ and *V*_2nd neighbour_ are scaled between 0 and 1, the GNN is able to distinguish between “atomic” and “environmental” (including bonding, see below) features of each atom, as contained in vector *X*_i_. The feature vector *X*_i_ was processed by one fully connected neural network layer, which was then connected to the message passing layer:2
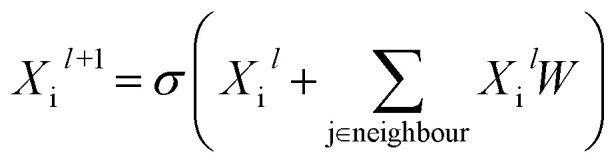
where *W* is the learnable parameter, *X*_i_^*l*^ is the representation of atom i at the *l*^th^ message passing layer, and *σ* is the rectified linear unit activation function.

Explicit bonding features were ignored in our featurisation as we believe that the detailed information about the type and valence of the atoms involved captures all relevant bonding information implicitly without further redundancy and ambiguity of the ease of bond rotation in various functional groups.

**Fig. 3:**
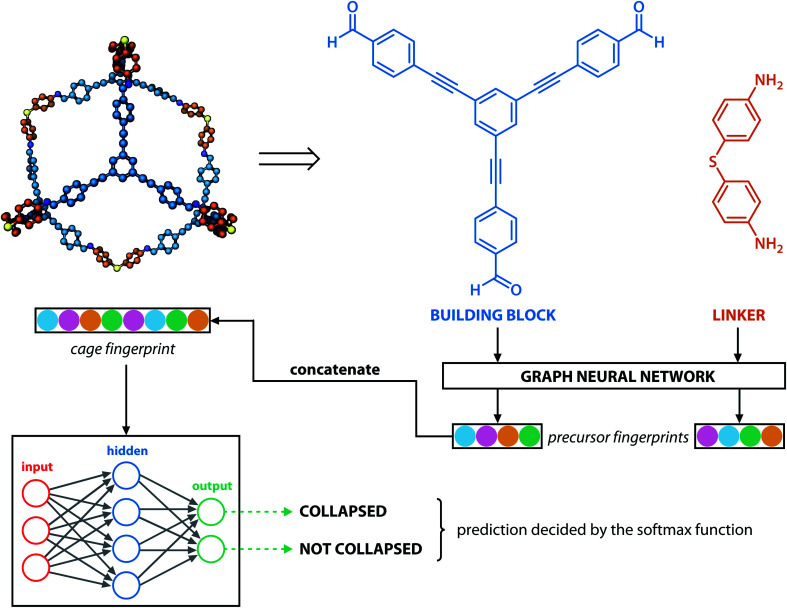
Architecture of the GNN in this study: monomers (building blocks and linkers) of the organic cages were encoded to numeric vectors using a graph neural network (see Fig. 2), the vectors were then concatenated and processed by a multi-layer neural network to output a shape persistence prediction. The prediction by the two neurons in the output layers was processed using the softmax function to obtain the final classification.

Similar to our previous work,^21^ the neural fingerprints for the organic cages in this study were obtained by concatenating the molecular vectors of the building blocks and linkers. Such neural fingerprints were then processed by a multi-layer neural network followed by a prediction layer (see Fig. 3). Parameters for the multi-layer neural network and the GNN were updated during the training process. As a result, neural fingerprints of the cage components were also updated. We used cross-entropy loss as the loss function and it operates with *C* output neurons for *C* classes, which can then be directly used for the corresponding prediction. Hence, the architecture of the prediction layer is determined by the predictive task in this study: for the classification tasks, such as predicting the organic cage shape persistence, the output layer has two neurons *z*_*i*_ (*i* = 1, 2). Each of the two output neurons was interpreted as the organic cage being “collapsed” or “not collapsed”, which were processed using the softmax function:3
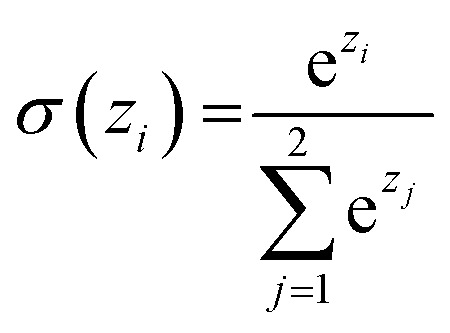


The neuron with the larger softmax output *σ*(*z*_*i*_) would be treated as the “predicted” label.

### 2.4 Training and evaluating the GNN models

In this study, we focused primarily on the classification GNN model, where the building block and linker of the organic cages were represented using GNN encoding, and the encoded vectors for the building block and linker molecules were concatenated so as to form a feature vector of the organic cage. The feature vector is then processed through a multi-layer neural network to predict the shape persistence of the organic cages. To examine the predictive power as well as the generalisability of the GNN models, two types of prediction tasks were employed. For the All-vs-One task, cross-reaction prediction was performed: the “collapsed” and “not collapsed” data in all but one row in Table 1 were used as the training set, and data in the remaining row were used as the test set. All rows in Table 1 were used iteratively for the All-vs-One task. For the All-vs-All task, the data for “collapsed” and “not collapsed” cages in Table 1 were randomly split to the training (80%) and test (20%) set. Performance of the All-vs-One model is an indicator of how transferrable the GNN model is towards cages generated *via* different reaction chemistries.

The performance of the GNN model on the classification task of “collapsed” and “not collapsed” cages was evaluated using the accuracy, precision and recall scores on the test sets, defined as follows:4
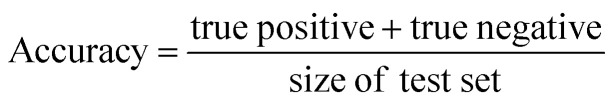
5
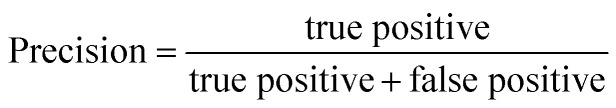
6
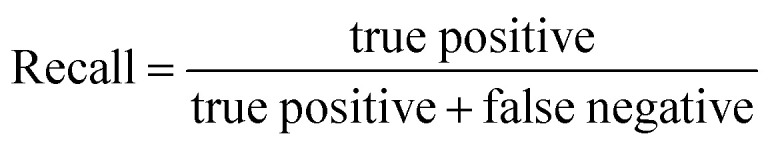


In this study, the “collapsed” organic cages were regarded as “positive” in our predictions. “True positive” represents the data where cages were “collapsed” from both the GNN model prediction and as labelled in the database; “false positive” represents the data where cages were “collapsed” according to the GNN model prediction but “not collapsed” as labelled in the database; “true negative” represents the data where cages were “not collapsed” from both the GNN prediction and as labelled in the database; “false negative” represents the cages that were “not collapsed” according to the GNN prediction but were “collapsed” as labelled in the database.

### 2.5 Explicability of the GNN models

**Fig. 4:**
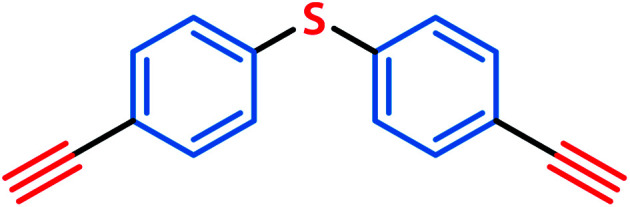
Example visualisation of per-atom contribution to the model prediction. Fragments with positive contributions (likely to contribute to pore collapse) are shown in red, while fragments with negative contributions (not likely to contribute to pore collapse) are shown in blue.

The explicability of the GNN model predictions was analysed by calculating the attribution score of the input features, which is the atomic input vectors to the GNN in this study. By calculating the attribution score, we aim to analyse which building block or linker molecules contribute more to the collapse of an organic cage, and which fragments in these molecules contribute more to the building block or linker being a “collapse-inducing” component of the cage. Collapse-inducing building blocks and linkers could be selected by simply looking at the prevalence of building blocks and linkers in the database, however, such a method does not consider the chemical structure of the precursors and is not transferrable to cages with precursors not included in the database. An example of per-atom contribution to the prediction is shown in Fig. 4, where the fragments with a positive attribution score (likely to contribute to pore collapse) are shown in red, and fragments with a negative attribution score (not likely to contribute to pore collapse) are shown in blue.

The attribution scores in this study were calculated and represented using integrated gradients. The formal definition for attribution scores, as well as the axiomatic justification of the integrated gradients satisfying certain properties is provided by Sundararajan *et al.*^26^ To explain briefly here, let function *F*:*R*^*n*^ → [0,1] represent a deep neural network. Given an input feature *x* and some baseline feature *x*′, the integrated gradient of *x* along the *i*^th^ dimension of *x* was defined as follows:7
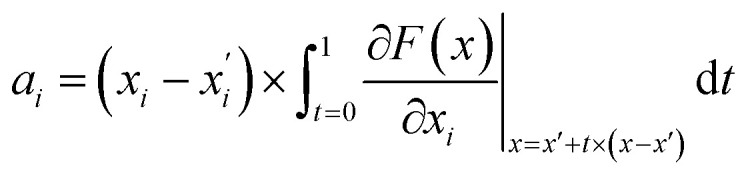
where 
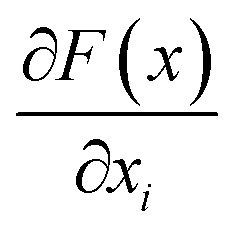
 is the gradient of *F* along the *i*^th^ dimension of *x*. In this study, the input *x* was the numeric vector for the organic cages, which is the concatenation of the feature vectors of building block and linker molecules, and *F* is the probability of the organic cage being “collapsed” as predicted by the GNN. This definition of integrated gradient is justified by the axiomatic result that it satisfies the requirement of *completeness*: the attributions of the input features (cage atoms) should add up to the difference between the output of *F* at the input *x* and the baseline *x′* for eqn (7).^26^ In this study, the probability of the organic cages and the corresponding baseline cages being “collapsed” were computed from the GNN models, the *completeness* of the attribution model requires that the difference between the two probability values Δ*P* should be equal to the integrated gradient of the input features for the organic cage *Σ*_ig_.

The integrated gradient attribution was defined relative to a baseline, and the selection of the baseline is essential to causal analysis of ML models.^31^ A robust baseline input should give uninformative predictions; for example, for a classification task, the ML model should give the probability of approximately 0.5 for the baseline input, which requires a comparable number of “collapsed” and “not collapsed” cages to mimic the distribution of *p* = 0.5 for cage collapse in the training set. Here, we used the input of zero vectors as the baseline molecule and augmented the training set using the baseline cages to achieve uninformative predictions for the baseline cages. The detailed implementation is provided in Section S2.†

Once the integrated gradients for all input atoms of the organic cage were calculated, the contribution of the building block and linker of the cage was calculated by summing up the integrated gradients for the atoms corresponding to the building block and linker, respectively. The attribution of fragments in the building block and linker molecules were visualised using the atomic integrated gradients in the molecules.

The GNN model, as well as the computation of integrated gradients were implemented in Python 3.7.5 combined with PyTorch 1.1.0; the source code is provided at http://github.com/qyuan7/Cage_GNN.

## 3. Results and discussion

### 3.1 Predictive performance of the GNN for organic cage shape persistence

A comparison of the predictive performance of the previously reported random forest model (used here as a benchmark) and the GNN model on the All-vs-All task is shown in Table 2, where the data for all cages in this study were randomly split to training and test sets. The GNN model and the random forest benchmark have comparable performance for the All-vs-All task, with the GNN model slightly outperforming the random forest model based upon the accuracy and precision metrics. The reason for the almost equally good performance of the GNN and random forest models on the All-vs-All task could originate from the dataset in this study. The building block and linker molecules in this study were built by changing the functional groups on a fixed set of precursor cores, and each row of organic cages in Table 1 was generated from only 118 unique ditopic precursors and 51 unique tritopic precursors. For the All-vs-All task, the dataset of all the organic cages in Table 1 was randomly split between the training and test sets, and the same precursor would possibly be present in both the training and test sets. In addition, for both the GNN and random forest models, the organic cages were represented by concatenating the molecular vectors of the precursors. It is therefore possible for both models to learn the “possibility” of a certain precursor belonging to a collapsed cage from the training set and to then make predictions on the test set. In this sense, both the GNN and the random forest learnt the probability distribution of certain precursors resulting in collapsed cages across all sets, but it is unclear how much of the contribution of the precursor towards cage shape persistence was learnt in the All-vs-All task. Therefore, the advantage of the neural fingerprints learnt from the GNN model of being more flexible is minimised in the All-vs-All task.

**Table 2:** Shape persistence prediction of the GNN and random forest models on the All-vs-All task. The models with better performance for each metric are highlighted in bold

	GNN	Random forest
Accuracy	**0.89**	0.88
Precision	**0.90**	0.89
Recall	0.90	**0.91**

The All-vs-One task, where data for cages in all but one row in Table 1 was used as the training set and the remaining row is used as the test set, is more challenging compared to the All-vs-All task, as most of the precursors in the test set were not included in the training set (except for the amine2 linkers and amine3 building blocks, which were used by two rows in Table 1). The All-vs-One task provides better evaluation of the transferability of the ML models towards different families of precursors with different functional groups, which carries more application significance for the design of future organic cages. The accuracy scores for the GNN and random forest models are shown in Table 3, and the corresponding precision and recall scores are provided in Table S2.† The results are for when a model was tested on a single data set within a row, *i.e.* with cages formed by a single reaction chemistry type. The data sets in the other rows were used as the training set. For example, for the test of aldehyde3amine2 cages (row 1), all the precursor pairs in the other rows were used as the training set (amine3aldehyde2, alkene3alkene2, *etc.*), only the aldehyde3amine2 cages were used as the test set.

**Table 3:** Shape persistence prediction of the GNN and random forest models on the All-vs-One task^a^

Building block	Linker	Test accuracy (Random forest)	Test accuracy (GNN)
Aldehyde3	Amine2	0.61	**0.72**
Amine3	Aldehyde2	0.72	**0.73**
Alkene3	Alkene2	0.63	**0.81**
Alkyne3	Alkyne2	0.41	**0.77**
Acid3	Amine2	0.71	**0.76**
Amine3	Acid2	0.73	**0.79**
Aggregated score (95% CI)		0.64 ± 0.10	**0.76 ± 0.03**

a Model with better performance for each task is highlighted in bold.

For the All-vs-One task, the GNN model consistently outperformed the random forest model and by a larger margin compared to the All-vs-All task. The biggest improvement in the predictive performance of the GNN model compared to the random forest benchmark was for the alkene3alkene2 cages (alkene metathesis of a trialkene and dialkene) and the alkyne3alkyne2 cages (alkyne metathesis of a trialkyne and dialkyne). As shown in Table 1, the building blocks for alkene3alkene2 and alkyne3alkyne2 cages were not used for the other cages, and the benchmark random forest model failed to give reasonably accurate predictions on the shape persistence of the alkene3alkene2 and alkyne3alkyne2 cages, thus the transferability for the benchmark random forest model is poor to building blocks that were not used in the training sets. The GNN model, on the other hand, was equally accurate for the predictions of the alkene3alkene2 and alkyne3alkyne2 cages compared to the other groups of cages. The consistent improvement in predictive power of the GNN model compared to the random forest model indicates that the GNN model has better transferability to novel precursors for cages and different reaction types. In addition, the improved performance of the GNN model for the alkene3alkene2 and alkyne2alkyne2 cages suggests that the GNN model has learnt some structural features of the precursors that led to collapse from the training process, providing the model with some “chemical intuition”, which can be investigated further by trying to explain and interpret the predictions of the GNN model using the integrated gradients.

It is worth discussing here possible extension to different cage topologies. In this work, we focused on the Tri^4^Di^6^ topology, which is only one of the six topologies resulting from a condensation of tritopic and ditopic precursors that we have previously enumerated.^28^ We foresee re-employment of our approach to cages in other topologies, not even limited to tri- and ditopic precursors. As it is difficult to predict *a priori* which topology will be preferred, we would recommend training separate models for other possible topologies using the same cage representation as the one used in this work (see Fig. 3). However, in the future we hope for a more general model, which would include neurons in the output layer containing probabilities of cage collapse in all possible topologies alongside a prediction of which topology is the thermodynamically more stable reaction outcome.

### 3.2 Explicability of the GNN predictions

**Fig. 5:**
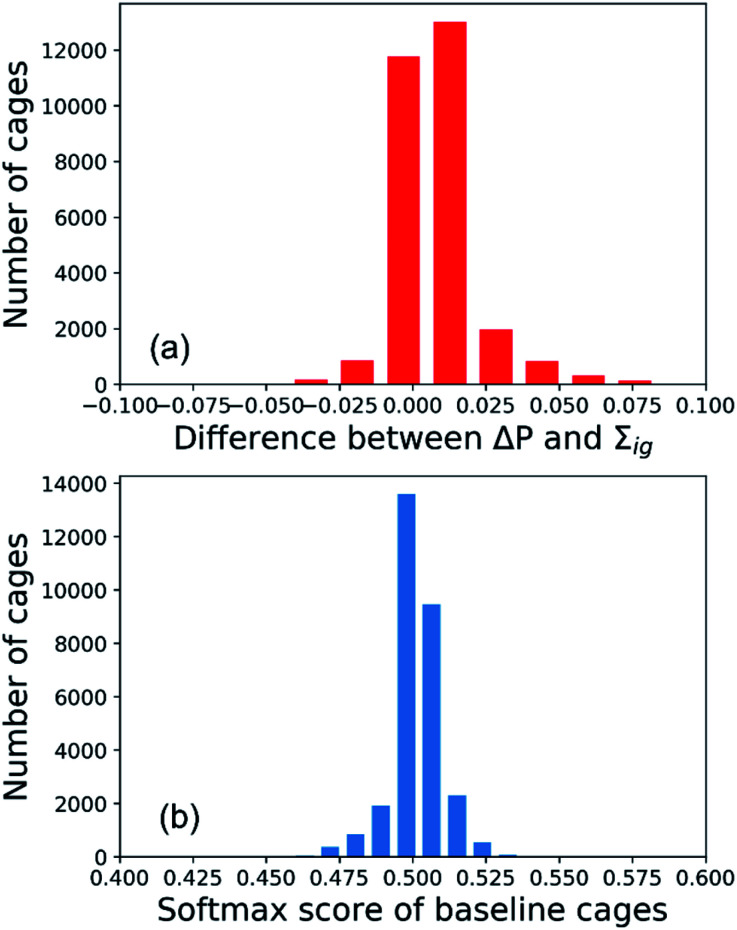
Validation of the integrated gradient calculations in this study: (a) distribution of the difference between the Δ*P* and *Σ*_ig_ values for all the cages in the test sets of the All-vs-One task; (b) distribution of the predicted softmax score of baseline cages.

To interpret the predictions of the GNN models for the All-vs-One task, we computed the integrated gradients of input vectors to the GNN, which were further summed up to get the integrated gradients of cage precursors and fragments in the test sets. Before analysing the results, we validated our calculations by checking the completeness of the integrated gradient in this study. The probability of the organic cages and the corresponding baseline cages being “collapsed” were computed from the GNN models, and the difference of the two probability values is noted as Δ*P*. The sum of integrated gradient of the input features of the organic cages is noted as *Σ*_ig_. The distribution of the difference between the Δ*P* and *Σ*_ig_ values for all the cages in the test sets of the All-vs-One task is shown in Fig. 5(a). The distribution is centred around 0, with a mean value of 0.008 and standard deviation of 0.013, indicating that the integrated gradient computed in this study meets the requirement of completeness for an attribution model.

In this study, the integrated gradient of the cage input feature *x* for an atom is defined relative to the baseline input *x*′ in eqn (7), thus it is important that the GNN model *F* should give uninformative predictions to the baseline input. For the classification task in this study, the baseline input should render a probability close to 0.5, indicating the baseline cage composed of vector of zeros should have neutralised probability of being “collapsed”. When calculating the integrated gradients, we used the data augmentation technique on the training set, as described in Section S2 of the ESI† and the work by McCloskey *et al.*^27^ The distribution of the predicted softmax scores for the “collapsed” neuron in the output layer (which can be interpreted as the probability of the cage being collapsed) on the baseline cages used in training the GNN model for calculating the integrated gradient is shown in Fig. 5(b). The softmax score of the baseline cages centres around 0.5 with a mean value of 0.501 and standard deviation of 0.008. This result indicates that the GNN model gives neutral predictions to the baseline cages, and for a cage with softmax score larger than 0.5 for the “collapsed” neuron in the output layer that is classified to be “collapsed”, the majority of the attribution to the increased softmax score can be ascribed to the molecular features of the building block and linker molecules of the cage.

### 3.3 Explicability of the GNN models – precursors with the highest integrated gradients

With the validation of the integrated gradient calculations completed, it was possible to calculate the attributions of the cage building blocks and the linkers and identify the precursors with a high integrated gradient contribution for the “collapsed” predictions. If some precursors have high integrated gradient scores in “collapsed” cages, it is possible that such precursors can be regarded as the “collapse-inducing precursors” that should be avoided in the design of novel organic cages. However, if the precursors' integrated gradient attribution scores have no strong correlation with the shape persistence, then the structural features of the “collapsed” precursors have not been learnt.

**Fig. 6:**
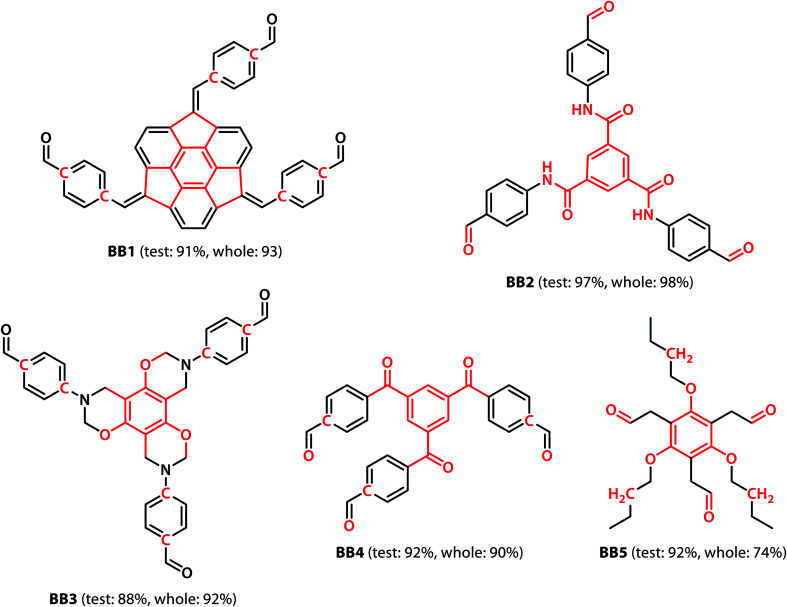
The top 5 building blocks with the largest overall integrated gradient attributions for the aldehyde3amine2 cages. Atoms with integrated gradients greater than 0.01 are highlighted in red. Percentages of cages containing each building block identified as “collapsed” in the test set and the highlighted backbones in the whole database are shown. The building blocks can be regarded as collapse-inducing precursors if they tend to form cages with high probability of collapse. The highlighted fragments in the building blocks are those contributing most to the integrated gradient of the corresponding building block.

**Fig. 7:**
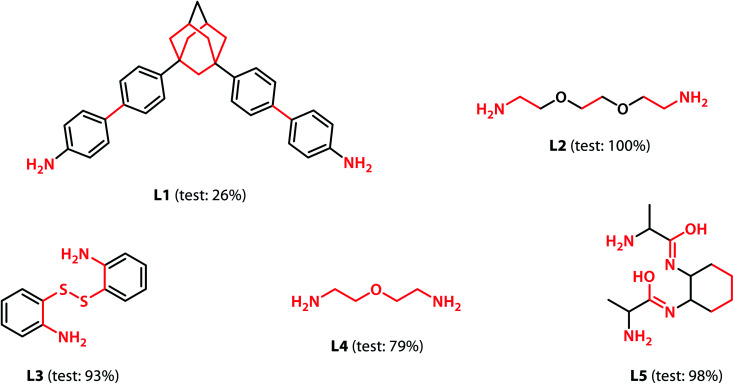
The top 5 linkers with the largest integrated gradient attributions for the aldehyde3amine2 cages. Atoms with integrated gradients greater than 0.01 are highlighted in red. Percentages of cages containing each building block identified as “collapsed” in the test set are shown. The linkers can be regarded as collapse-inducing precursors if they tend to form cages with high probability of collapse. The highlighted fragments in the linkers are those contributing most to the integrated gradient of the corresponding building block.

We calculated the integrated gradients of the precursors in the test sets for the All-vs-One tasks and ranked the building blocks and linkers according to their integrated gradient attribution scores. The top 5 building blocks BB1–5 for the cages generated from the aldehyde3amine2 (imine reaction of trialdehyde and diamine) cages with the largest integrated gradient are shown in Fig. 6. The percentage of aldehyde3amine2 cages containing the building blocks that were “collapsed” in the All-vs-One test set are also shown. It can be seen that almost all the building blocks in Fig. 6 have a probability of larger than 90% of being “collapsed”, indicating that cages with these building blocks have a great chance of being “collapsed” and that these building blocks should be avoided in the design of future organic cages for the sake of shape persistence. The top 5 linker molecules L1–5 for the aldehyde3amine2 cages with the largest integrated gradient attribution to “collapsed” cages are shown in Fig. 7, with the percentage of “collapsed” aldehyde3amine2 cages containing the linker molecules shown. Apart from L1, the cages in the test set containing these linkers have a high probability of being “collapsed”. The integrated gradients of the building block and linker molecules can thus serve as an indicator for the organic cages being “collapsed” – using building block/linker molecules with high integrated gradient attributions means there is a high probability of collapsed cages. It might be tempting to assume that precursors with smallest gradient attributions would indicate “non collapsed” cages. The “bottom 5” building blocks and linkers of the aldehyde3amine2 cages are shown in Fig. S11 and S12.† Cages with such building blocks and linkers still have a considerable possibility of being “collapsed”, thus the integrated gradient has only limited effect of identifying less collapse-inducing precursors. Limitation of the GNN model in identifying “non collapsed” cages could also be observed from the low specificity scores for the All-vs-One tasks, as shown in Table S2.† We, therefore, focus on the collapse-inducing precursors in this study.

The top building blocks and linkers for the other groups of organic cages with the largest integrated gradient together with the probability of a cage being “collapsed” with such precursors are provided in Section S4 of the ESI.† For the acid3amine2 cages (amide condensation of a tricarboxylic acid and diamine), the integrated gradient attributions of the top building blocks had poor correlation to the cage shape persistence, which could be because the carboxylic acid functional group was used less in the database (Table 1), and the GNN model therefore had poorer transferability to the cages with the tricarboxylic acid building blocks. Further improvement of the GNN model for the cages formed *via* amide condensation reaction would require a larger dataset labelled as per the current dataset. The relationship of cage shape persistence and the average integrated gradient attribution scores for the building block/linker molecules in the All-vs-One task is shown in Fig. S11 and S12.† Qualitative agreement of cage collapse and high integrated gradient scores can be found for cages formed *via* imine condensation, alkene metathesis and alkyne metathesis, which could provide initial insight into the shape persistence of organic cages formed *via* such reaction chemistries (see Fig. S13 and S14†).

If specific precursor fragments could be identified as collapse-inducing from the above analysis, then such a fragment could be usefully avoided in the design of novel precursors. Atoms in the cage components with an integrated gradient attribution score greater than 0.01 are highlighted in red in Fig. 6 and 7. The majority of the integrated gradient attribution is located in the central core of the building blocks; and such fragments could contribute to the poor shape persistence of the corresponding cages. It is thus possible to identify molecular fragments/centre cores that have high attribution to the collapse of organic cages and to therefore avoid/alter such fragments when selecting precursors for cage synthesis. In order to validate whether the identified central cores correlate with the shape persistence of all the organic cages in this study, we performed a sub-structure match of the cores across all the cages in this study and calculated the probability of a cage with precursors containing the backbones being collapsed, which is also shown in Fig. 6. Meanwhile, the linkers in Fig. 7 (apart from the outlier L1) contain more saturated carbon chains and hence more internal degrees of freedom. Furthermore, the amine part of the imine bond (resulting from the condensation to give cages in the aldehyde3amine2 set) contains one more flexible methylene unit compared to the aldehyde contribution. As a result, the fragments with high integrated gradients for linkers L2–5 span over both the linker backbone and the functional group, making it difficult to attribute the GNN prediction to any particular motif within those molecules, and therefore substructure matching of the linker molecules was not performed.

**Fig. 8:**
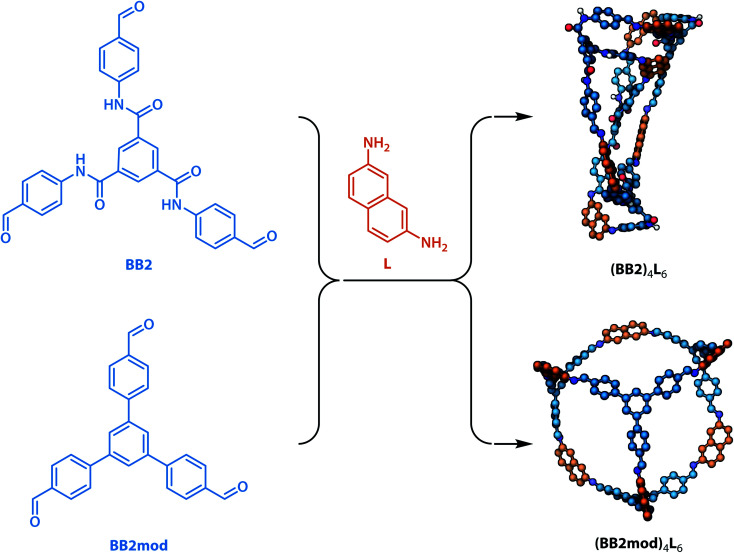
Proof-of-concept replacement of the collapse-inducing core in building block BB2 with a less collapse-inducing unit in the modified BB2mod. Upon imine formation with the same linker L and geometry optimisation, cage (BB2)_4_L_6_ was found to be “collapsed” (as expected from the GNN model) and cage (BB2mod)_4_L_6_ exhibited significantly more open structure (improved shape persistence). In 3D models, non-polar hydrogen atoms are omitted for clarity, carbon atoms are based on whether they originate from the building block (blue) or the precursor (orange), nitrogen atoms are shown in dark blue, oxygen atoms are shown in red.

To investigate whether the integrated gradient analysis can help chemists design cages with improved shape persistence, we replaced the collapse-inducing core of building block BB2 with a simple rigid benzene ring (yielding BB2mod). After identical MD geometry optimisation conditions as those used in the training set, the modification provided a shape persistent cavity (see Fig. 8). This demonstrates that the GNN model not only shows higher accuracy than the previously reported models but also that the integrated gradients analysis is a powerful tool for molecular design.

## 4. Conclusions

We developed graph neural network (GNN) models to predict the shape persistence of organic cages computationally generated *via* a range of reactions. The GNN model has better performance compared to our previously published random forest model,^21^ especially for cross-reaction prediction tasks. Apart from the improved predictive performance, we evaluated the explicability of the GNN models by computing the precursor-wise and atom-wise integrated gradients. We showed that integrated gradients can be used to learn structural features of the precursors that contribute to the collapse of organic cages, which could help exclude precursors that are more likely to result in collapsed cages. For the generally more rigid building blocks, the core backbones appear to be of greatest importance for collapse prediction, while for the smaller and more flexible linker molecules, the collapsibility appears to originate from saturated aliphatic chains and the corresponding increased degrees of freedom, as would be expected.

The computational study of supramolecular systems such as organic cages is time consuming using physical simulations, and the development of ML techniques has the potential to provide data-driven solutions that might accelerate the evaluation of supramolecular systems. However, in many cases the ML models are regarded as powerful black-boxes, providing limited insight to further the materials discovery process further. In this study, we aimed to develop an explainable GNN model both to ensure the transferability of our model and to provide guidance for further material discovery.

## Data availability

All the data, code and models in this study is available in the Github repository https://github.com/qyuan7/Cage_GNN. Structural and shape persistence data of all cages in this study is available in file filtered_all_smi.csv following the link. In order to process the information to input vectors to the GNN model, run the code database/db_preparation.py. Models trained in this study are available in the trained_models/folder. Detailed descriptions and further information can be found in the above Github repository.

## Conflicts of interest

There are no conflicts to declare.

## Supplementary Material

DD-001-D1DD00039J-s001
