# Supplementary material for: Explainable graph neural networks for organic cages
Source: Digit Discov. 2022 Feb 11;1(2):127–38. doi: 10.1039/d1dd00039j (PMC8996732; doi:10.1039/d1dd00039j)
Supplement: DD-001-D1DD00039J-s001 [file DD-001-D1DD00039J-s001.pdf]

Electronic Supplementary Material (ESI) for Digital Discovery.
This journal is © The Royal Society of Chemistry 2022

Explainable Graph Neural Networks for Organic Cages

Supporting Information

Qi Yuan, Filip T. Szczypiński, and Kim E. Jelfs*

Department of Chemistry, Molecular Sciences Research Hub, White City Campus, Imperial College London, Wood Lane, London, UK

*E-mail address: k.jelfs@imperial.ac.uk

Contents

S1.Cage collapse labels for the different reactions in this study. 3

S2. Integrated gradient baseline..3

S3. Detailed performance comparison of GNN and random forest for the All-vs-One task...................4

S4. Visualization of the integrated gradient attributions of precursor and fragments 4

S5. References 13

S1. Cage collapse labels for the different reactions

Table S1 Summary of computational label of organic cages in this study. The absolute number and (percentages) with each label are shown in the last three columns.

building block	linker	Collapsed	Shape persistent	Undetermined
aldehyde 3	amine 2	2269 (38%)	2314 (38%)	1435 (24%)
amine 3	aldehyde 2	2445 (40%)	2206 (37%)	1367 (23%)
alkene 3	alkene 2	2529 (42%)	2026 (34%)	1463 (24%)
alkyne 3	alkyne 2	1981 (33%)	3148 (52%)	889 (15%)
carboxylic acid 3	amine 2	3973 (66%)	1105 (18%)	940 (16%)
amine 3	carboxylic acid 2	3724 (62%)	1368 (23%)	926 (15%)

S2. Integrated gradient baseline

The baseline cage molecule is an important part of the calculation of integrated gradient. Specifically, the GNN model has to give uninformative predictions to the baseline cages: for a binary classification task for cage shape persistency prediction (ML model assigns categorical labels '0' or '1' to an input cage), the GNN model should ideally give a predicted probability of approximately 0.5 for a dummy baseline cage to be "collapsed". In this study, we used vectors of zeros to represent the baseline cage, and adapted the data augmentation technique[1] to ensure that the GNN models give satisfies the above requirement.

The training set for integrated gradient computation was built by the GNN learnt neural fingerprints for cages with the correct labels (1 or 0), the baseline zero vectors for the same cages with label 1 ("collapsed"), and the baseline zero vectors for the same cages with label 0 ("not collapsed"). For each GNN model, the training set was sampled from the database so that the "collapsed" and "not collapsed" cages were equally numbered. This achieved the result that the baseline cages rendered neutral probability (~0.5) of being "collapsed".

S3. Performance comparison of GNN and random forest for the All-vs-One task

Table S2 Comparison of the GNN and random forest models on the All-vs-One tasks.

building block	linker	Precision-GNN	Recall GNN	Specificity GNN	Precision (RF)	Recall (RF)
aldehyde 3	amine 2	0.65	0.97	0.45	0.56	0.99
amine 3	aldehyde 2	0.67	0.96	0.37	0.66	0.94
alkene 3	alkene 2	0.83	0.82	0.76	0.61	0.96
alkyne 3	alkyne 2	0.64	0.96	0.56	0.40	1.00
carboxylic acid 3	amine 2	0.79	0.95	0.14	0.95	0.66
amine 3	carboxylic acid 2	0.86	0.79	0.36	0.94	0.67

S4. Visualization of the integrated gradient attributions of precursors and fragments

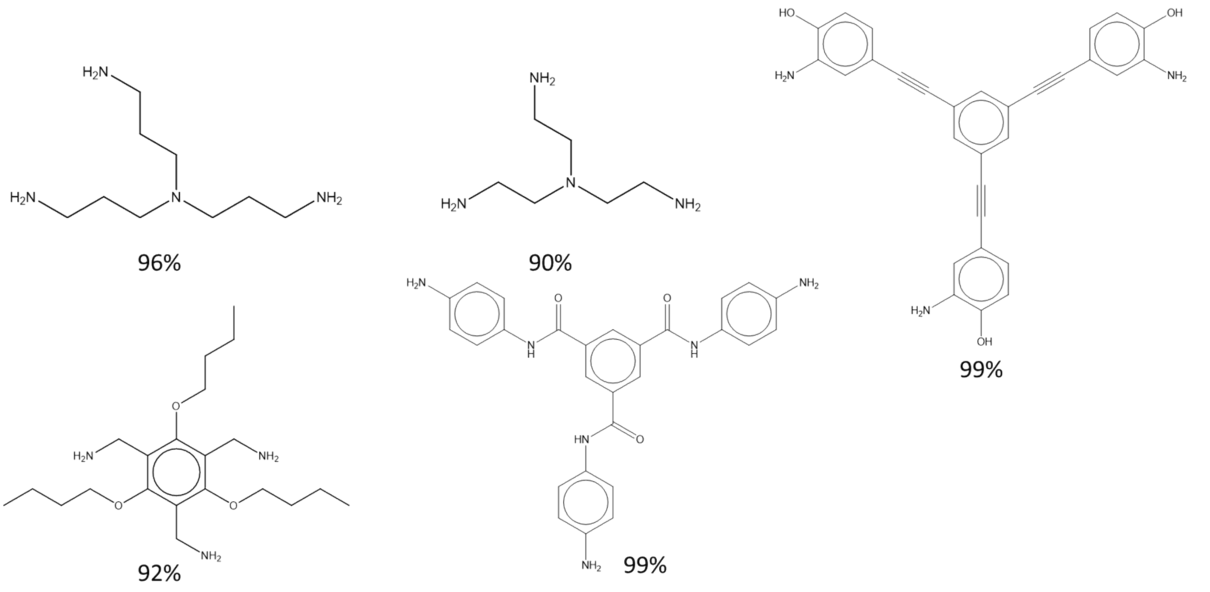

Figure S1 The top 5 building blocks with the largest integrated gradient attributions for the amine3aldehyde2 cages, the percentage of cages being "collapsed" in the test set is also shown.

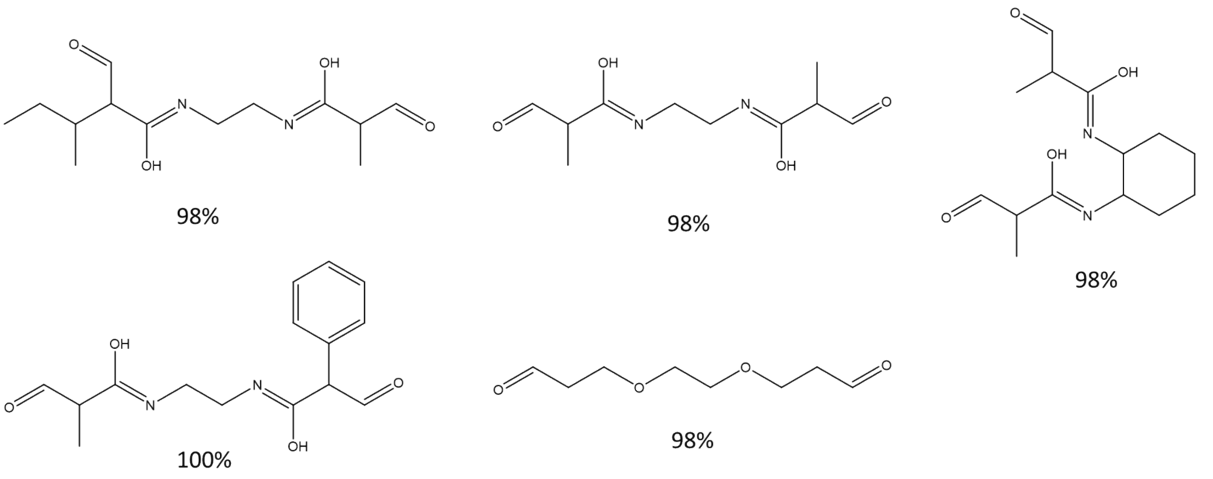

Figure S2 The top 5 linkers with the largest integrated gradient attributions for the amine3aldehyde2 cages, the percentage of cages being "collapsed" in the test set is also shown.

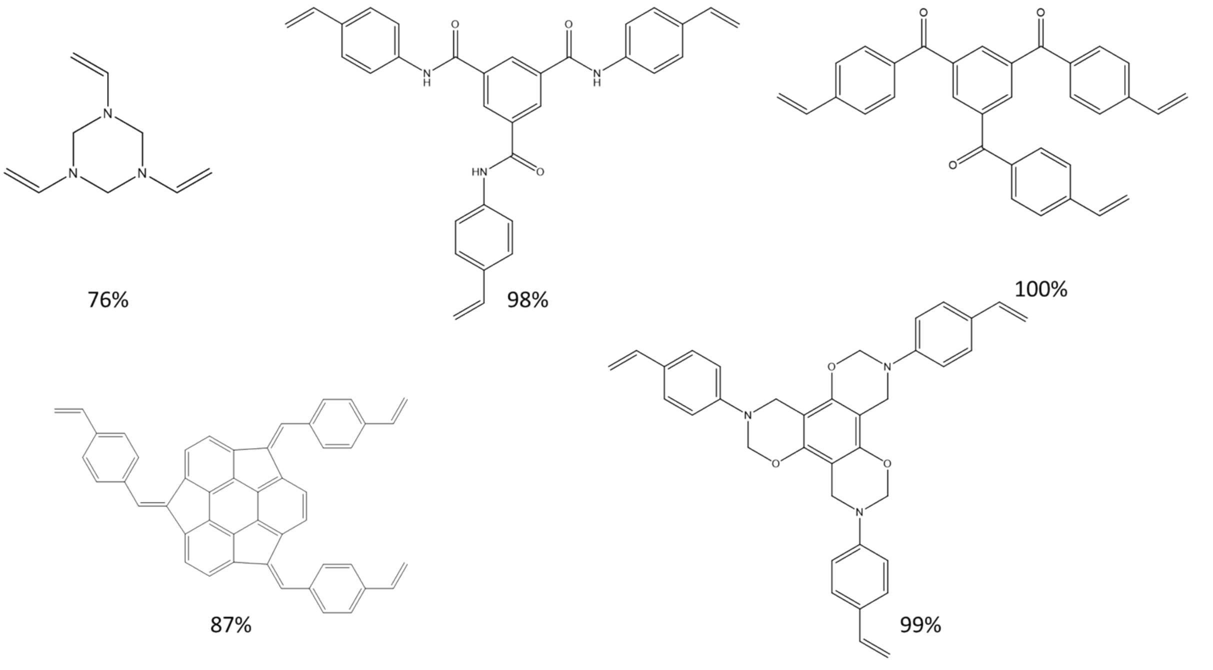

Figure S3 The top 5 building blocks with the largest integrated gradient attributions for the alkene3alkene2 cages, the percentage of cages being "collapsed" in the test set is also shown.

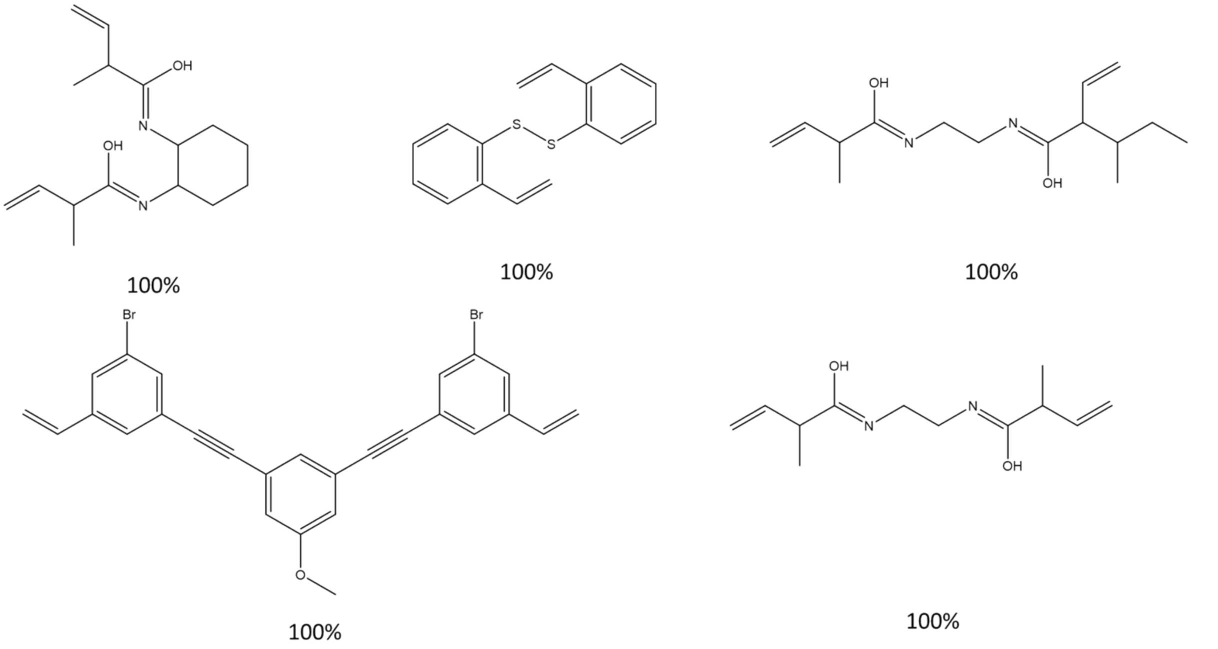

Figure S4 The top 5 linkers with the largest integrated gradient attributions for the alkene3alkene2 cages, the percentage of cages being "collapsed" in the test set is also shown.

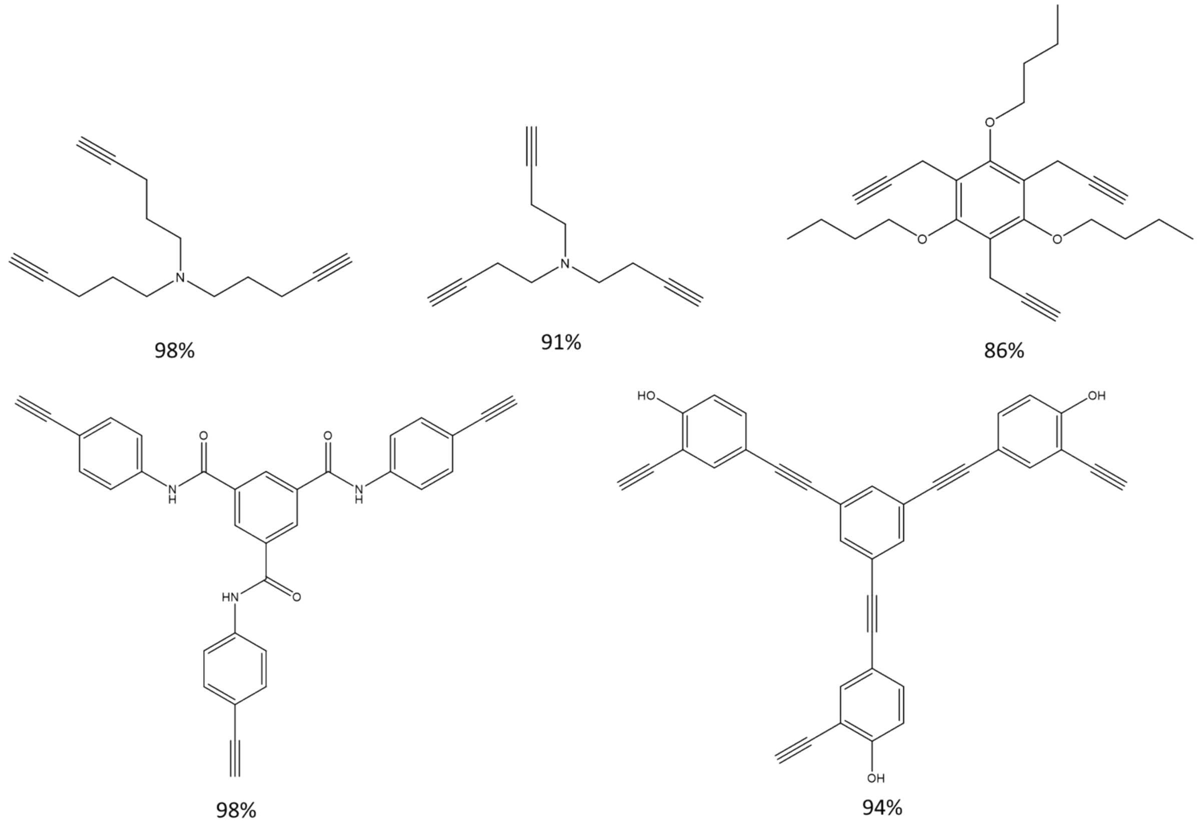

Figure S5 The top 5 building blocks with the largest integrated gradient attributions for the alkyne3alkyne2 cages, the percentage of cages being "collapsed" in the test set is also shown.

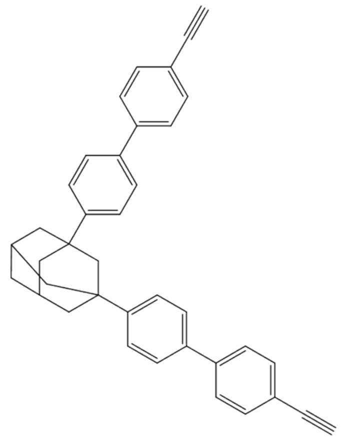

23%

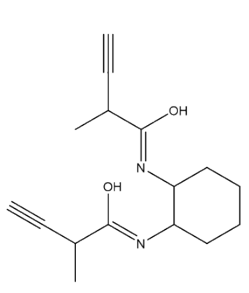

92%

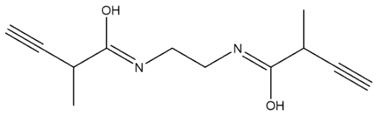

100%

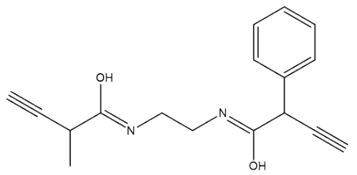

96%

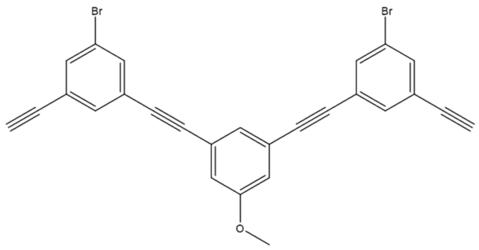

91%

Figure S6 The top 5 linkers with the largest integrated gradient attributions for the alkyne3alkyne2 cages, the percentage of cages being "collapsed" in the test set is also shown.

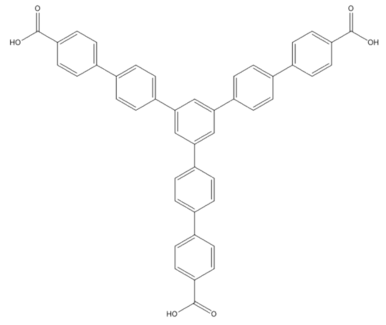

38%

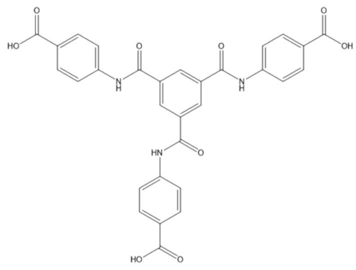

98%

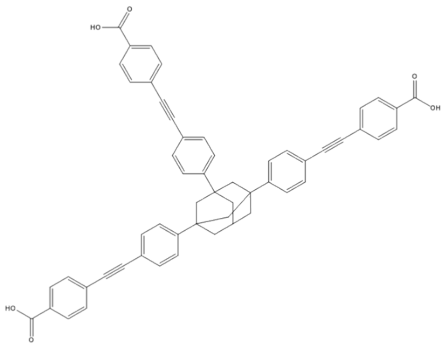

45%

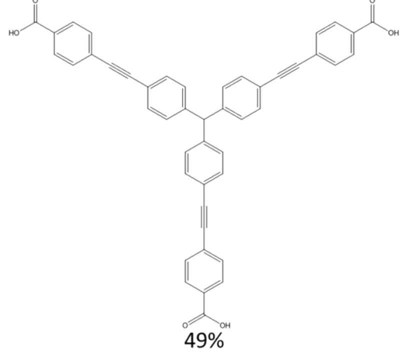

49%

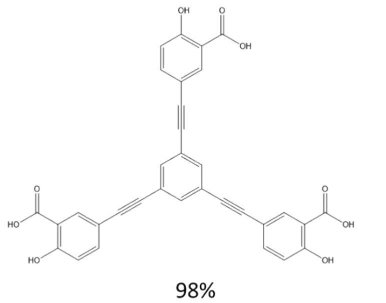

98%

Figure S7 The top 5 building blocks with the largest integrated gradient attributions for the carboxylicacid3amine2 cages, the percentage of cages being "collapsed" in the test set is also shown.

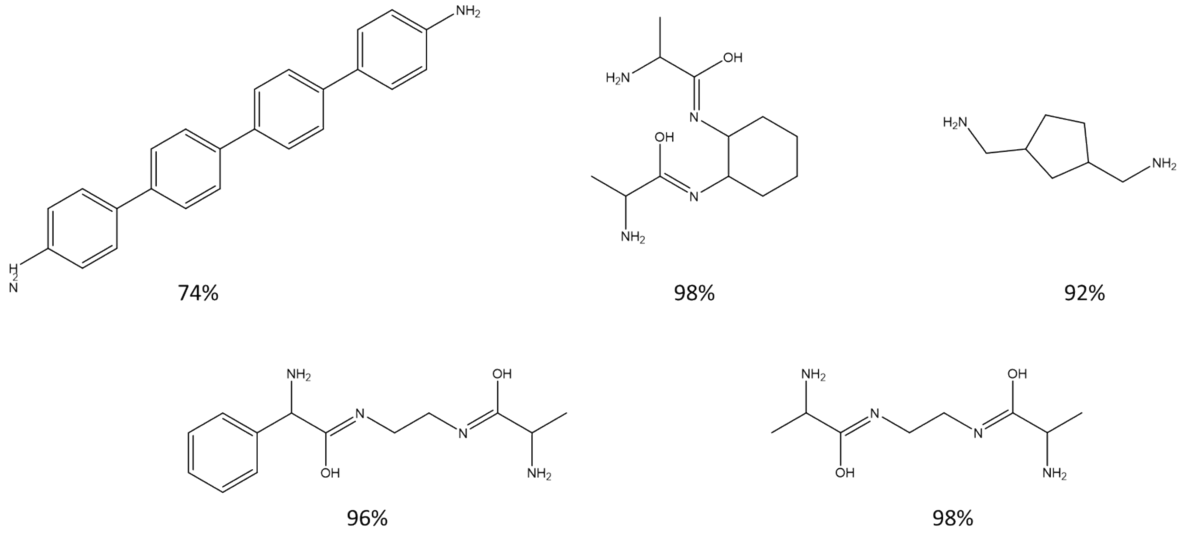

Figure S8 The top 5 linkers with the largest integrated gradient attributions for the carboxylicacid3amine2 cages, the percentage of cages being "collapsed" in the test set is also shown.

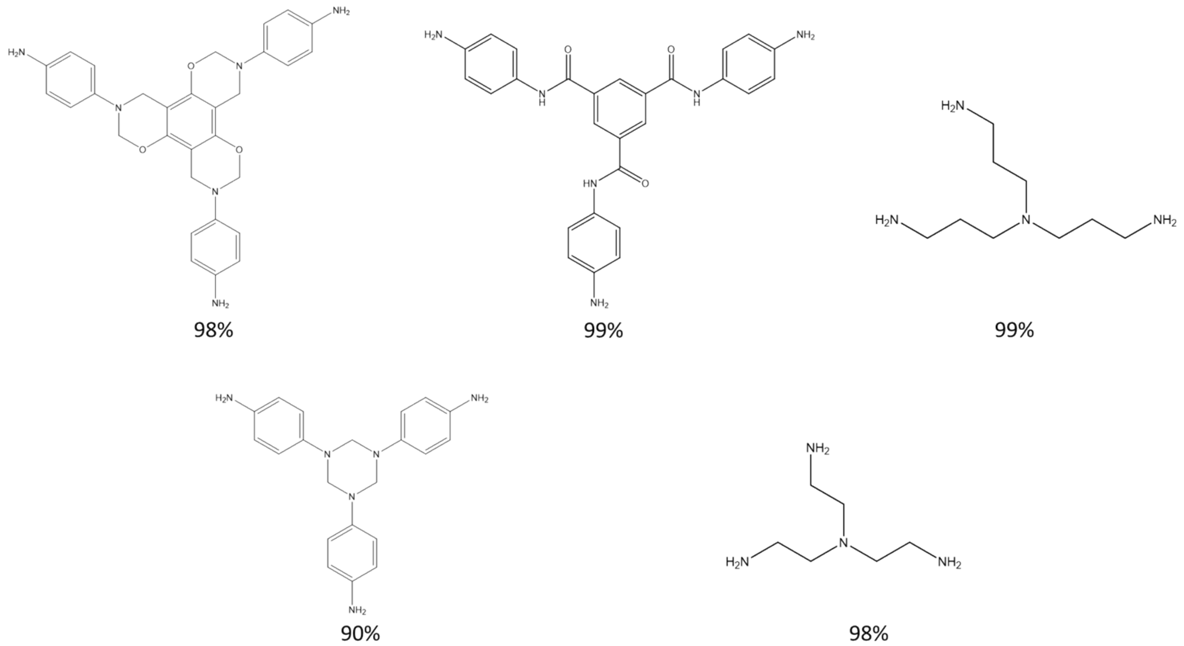

Figure S9 The top 5 building blocks with the largest integrated gradient attributions for the amine3carboxylicacid2 cages, the percentage of cages being "collapsed" in the test set is also shown.

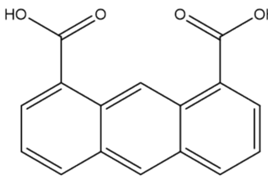

90%

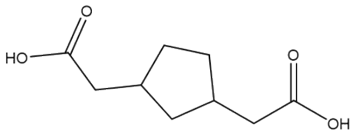

96%

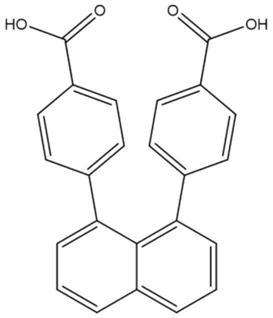

96%

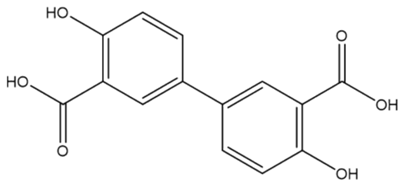

75%

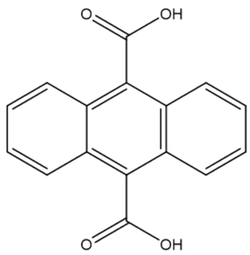

85%

Figure S10 The top 5 linkers with the largest integrated gradient attributions for the amine3carboxylicacid2 cages, the percentage of cages being "collapsed" in the test set is also shown.

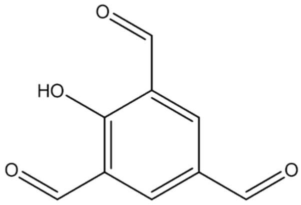

(a) 28%

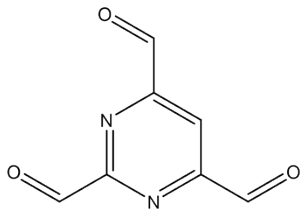

(b) 34%

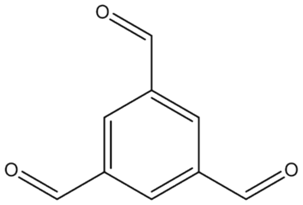

(c) 32%

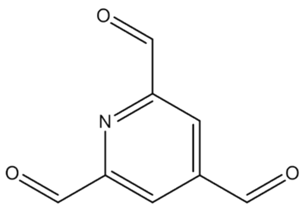

(d) 39%

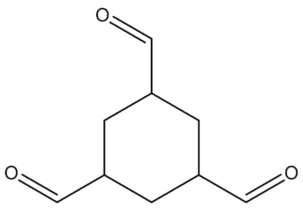

(e) 34%

Figure S11 The 5 building blocks with smallest integrated gradients for the aldehyde3amine2 cages, the percentage of cages being "collapsed" is also shown.

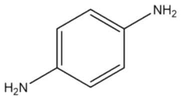

(a) 69%

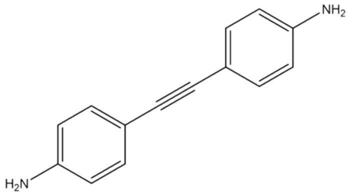

(b) 40%

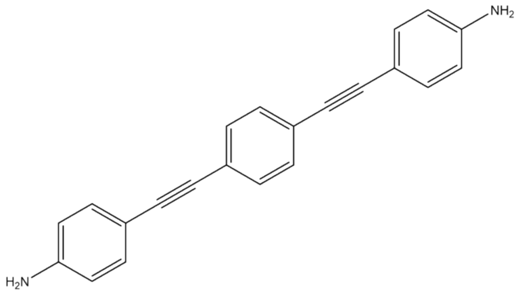

(c) 21%

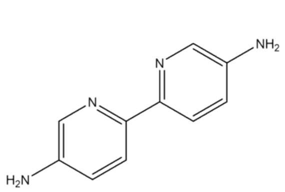

(d) 58%

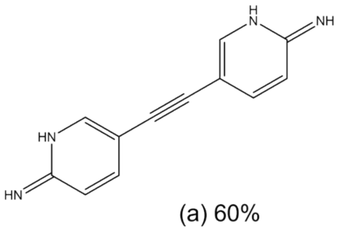

(a) 60%

Figure S12 The 5 building blocks with smallest integrated gradients for the aldehyde3amine2 cages, the percentage of cages being "collapsed" is also shown.

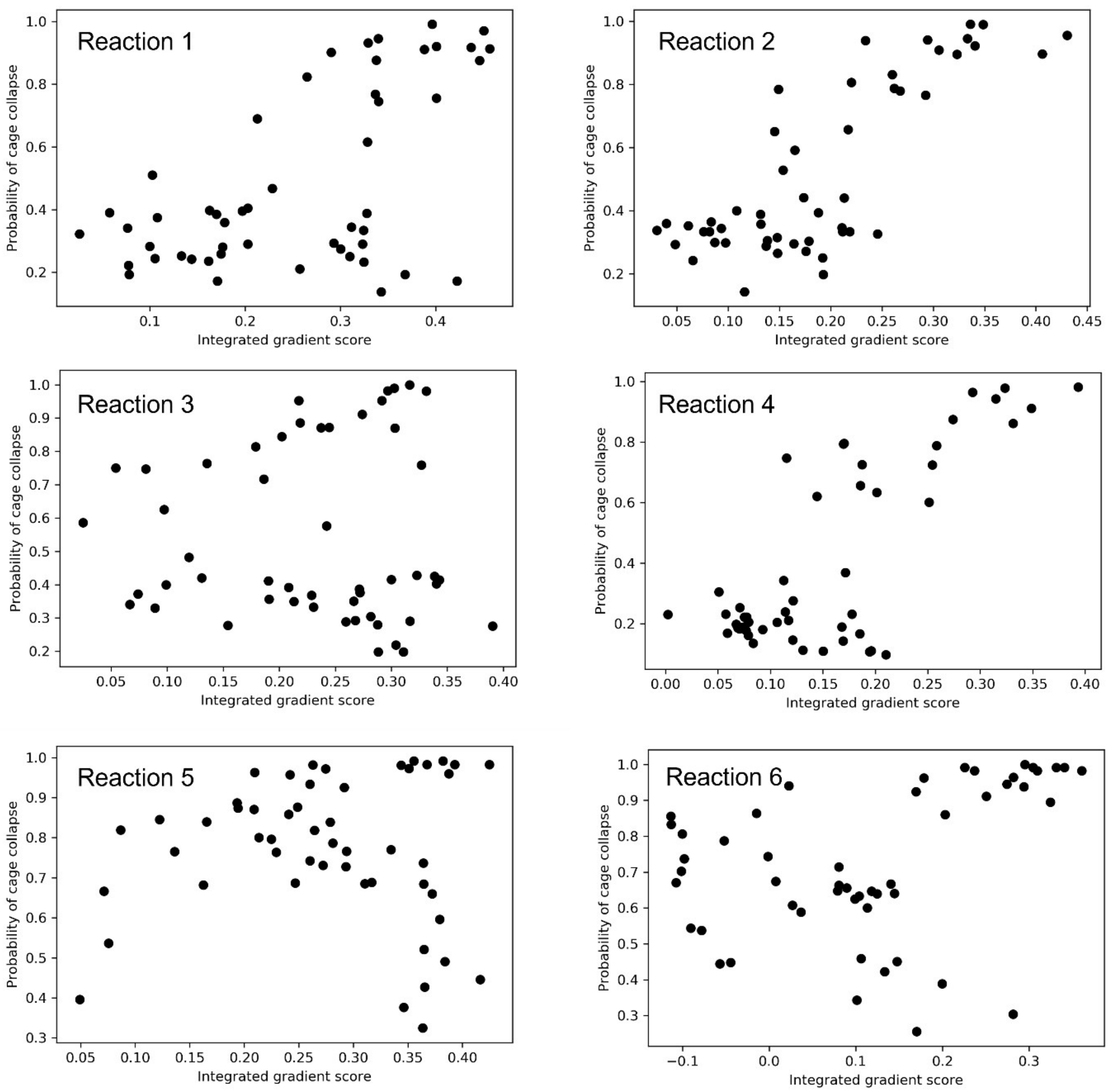

Figure S13 Relationship between the average integrated gradient score for the cage building blocks and the possibility of cages containing such building blocks being "collapsed" for the All-vs-One prediction tasks.

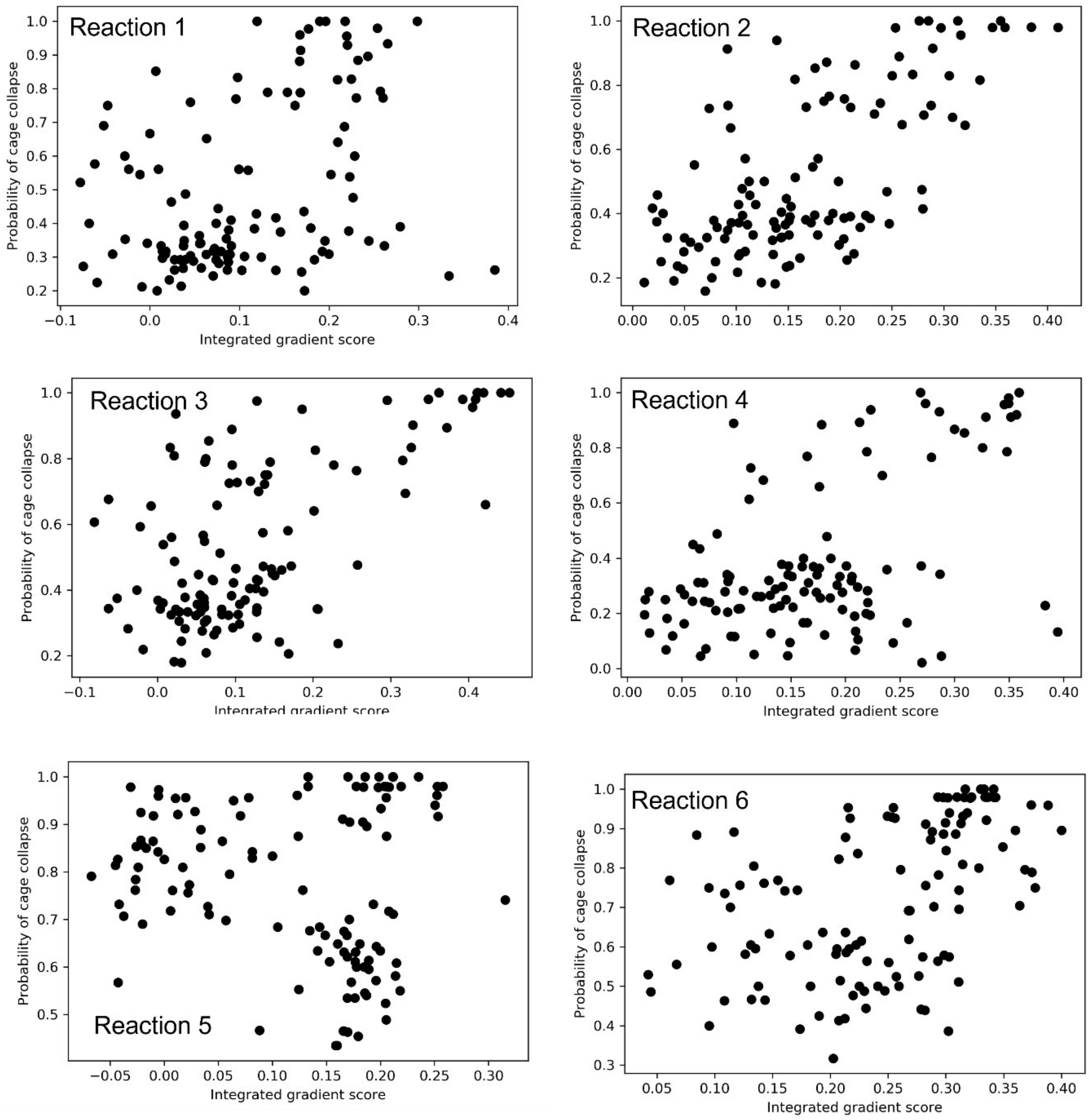

Figure S14 Relationship between the average integrated gradient score for the cage linkers and the possibility of cages containing such linkers being "collapsed" for the All-vs-One prediction tasks.

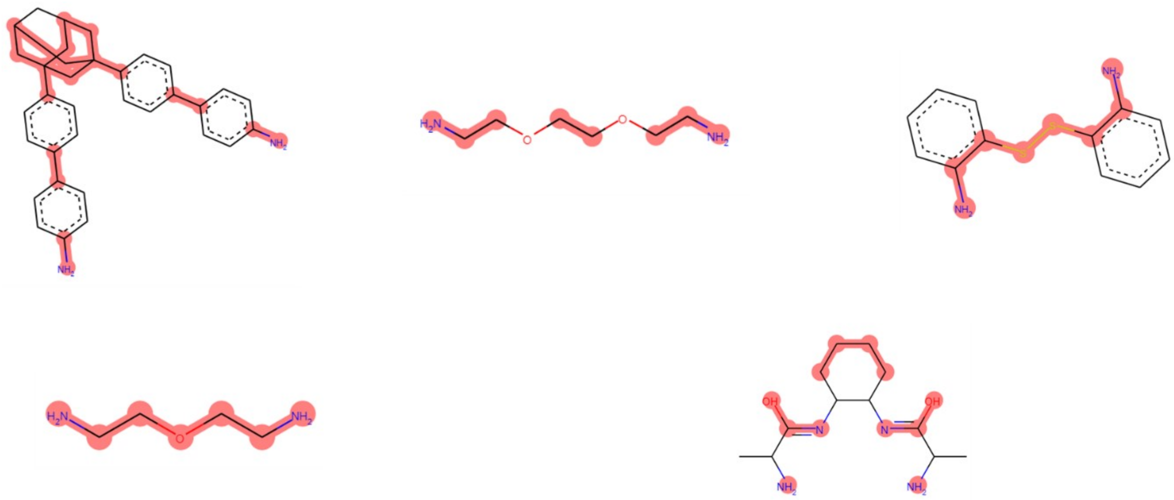

Figure S15 Top 5 linkers with highest integrated gradients for the aldehyde3amine2 cages in this study. The atoms with integrated gradient of greater than 0.01 are highlighted.

S5. References

[1] K. McCloskey, A. Taly, F. Monti, M.P. Brenner, L.J. Colwell, Using attribution to decode binding mechanism in neural network models for chemistry, Proc. Natl. Acad. Sci. 116 (2019) 11624–11629.
